# Automated drusen detection in retinal images using analytical modelling algorithms

**DOI:** 10.1186/1475-925X-10-59

**Published:** 2011-07-12

**Authors:** André D Mora, Pedro M Vieira, Ayyakkannu Manivannan, José M Fonseca

**Affiliations:** 1 Center of Technologies and Systems, Uninova, Campus da FCT-UNL, 2829-516 Caparica, Portugal; 2 Department of Electrotechnical Engineering, Faculty of Sciences and Technology, Universidade Nova de Lisboa, Campus da FCT-UNL, 2829-516 Caparica, Portugal; 3 Department of Physics, Faculty of Sciences and Technology, Universidade Nova de Lisboa, Campus da FCT-UNL, 2829-516 Caparica, Portugal; 4 NHS Grampian Department of Bio-Medical Physics and Bio-Engineering, NHS Grampian and University of Aberdeen, Aberdeen AB25 2ZD, Scotland, UK

## Abstract

**Background:**

Drusen are common features in the ageing macula associated with exudative Age-Related Macular Degeneration (ARMD). They are visible in retinal images and their quantitative analysis is important in the follow up of the ARMD. However, their evaluation is fastidious and difficult to reproduce when performed manually.

**Methods:**

This article proposes a methodology for Automatic Drusen Deposits Detection and quantification in Retinal Images (AD3RI) by using digital image processing techniques. It includes an image pre-processing method to correct the uneven illumination and to normalize the intensity contrast with smoothing splines. The drusen detection uses a gradient based segmentation algorithm that isolates drusen and provides basic drusen characterization to the modelling stage. The detected drusen are then fitted by Modified Gaussian functions, producing a model of the image that is used to evaluate the affected area.

Twenty two images were graded by eight experts, with the aid of a custom made software and compared with AD3RI. This comparison was based both on the total area and on the pixel-to-pixel analysis. The coefficient of variation, the intraclass correlation coefficient, the sensitivity, the specificity and the *kappa *coefficient were calculated.

**Results:**

The ground truth used in this study was the experts' average grading. In order to evaluate the proposed methodology three indicators were defined: AD3RI compared to the ground truth (A2G); each expert compared to the other experts (E2E) and a standard Global Threshold method compared to the ground truth (T2G).

The results obtained for the three indicators, A2G, E2E and T2G, were: coefficient of variation *28.8 *%, *22.5 *% and *41.1 *%, intraclass correlation coefficient *0.92, 0.88 *and *0.67*, sensitivity *0.68*, *0.67 *and *0.74*, specificity *0.96*, *0.97 *and *0.94*, and *kappa *coefficient *0.58*, *0.60 *and *0.49*, respectively.

**Conclusions:**

The gradings produced by AD3RI obtained an agreement with the ground truth similar to the experts (with a higher reproducibility) and significantly better than the Threshold Method. Despite the higher sensitivity of the Threshold method, explained by its over segmentation bias, it has lower specificity and lower *kappa *coefficient. Therefore, it can be concluded that AD3RI accurately quantifies drusen, using a reproducible method with benefits for ARMD evaluation and follow-up.

## Background

**Figure 1:**
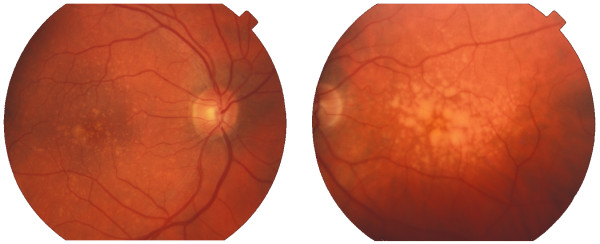
**Example of retinal images obtained by fundus photography**. In this figure two retinal images are shown. Left-side image contains hard drusen deposits (typically *< 63 μm *diameter yellow bright spots in and around the macula). Right-side image contains soft drusen deposits (typically *≥ 63 μm *and with smooth contours).

Drusen are considered as one of the Age-Related Macular Degeneration (ARMD) main risk factors [1]. These are retinal abnormalities, caused by the accumulation of extra-cellular materials beneath the retina surface. Despite that, on their own, they usually don't cause vision loss, although they can contribute to the development of ARMD. They are visible in retinal images as yellow round deposits that can be located anywhere in the retina (Figure 1). However, their consequences are more severe when located in the macula. The diagnosis and the follow up of drusen are commonly done through an evaluation of the affected area in fundus images. This evaluation, in a sequence of images taken during a long term treatment, helps to understand the progression of the disease and the effectiveness of the treatment. Nevertheless, this evaluation is fastidious and difficult to reproduce when performed manually. Therefore, the automation with digital image processing techniques, will enable the establishment of a stable criterion which in turn will certainly improve the follow up of this disease.

The automatic analysis of retinal images can be influenced by several factors. Misalignment between patient eye and camera, contracted pupil or cataracts can produce images with non-uniform illumination patterns, making retina analysis more difficult. The correction of the image contrast is an important step to improve the automatic processing of the retinal images. Histogram *equalization *and *specification *have been used to normalize retinal images contrast [2-4]. However, they are not able to correct localized non-uniformities. Smith *et al*. [5] presented a method to correct the contrast on the macular region, which obtained a good normalization, but required user intervention to specify the macula location and did not correct other illumination distortions.

Several studies for drusen segmentation have been published in the last twenty-years. Local thresholds [6-11], global thresholds [12] or fuzzy logic thresholds [13] were some of the proposed solutions for drusen segmentation. However, threshold techniques are significantly tampered by noise, requiring a good noise removal method.

In this work we propose a novel methodology for automated drusen detection and quantification that includes:

• an image pre-processing method to compensate the non-uniform illumination and to normalize the contrast [14];

• a detection method [15] to determine the number and location of drusen spots; and

• an image modelling method to quantify the affected area.

## Methods

### Materials

The retinal images which were used to validate the proposed methodology were collected from two collaborating research centres. Twenty two film images were selected, digitalized and saved as bitmap with 1000 × 1000 24-bit colour pixels. Eight experts (four ophthalmologists and four trained technicians) marked digitally the existing drusen using the application, *Manual Drusen Deposits Detection in Retinal Images *(*MD3RI*) that was specifically developed for this purpose [16] and made available on the internet [17]. This application allows computer assisted drawing of drusen contours, saving time and effort to the users and obtaining a very precise manual detection. In this study, the Wisconsin Grading System recommendations [18] were adopted. Following these recommendations, the inner-macula was defined as the *region of interest *(circular region of 3000 μm diameter around the Macula).

### Image processing

The proposed methodology defines all image processing steps to determine the area affected by drusen in order to establish a uniform analysis criterion. In the first processing step, the effects of non-uniform illumination are reduced and the contrast is normalized. The second processing step is the drusen detection, followed by the drusen modelling that detects and characterizes the drusen spots. On the fourth and last step, the affected area is quantified using the drusen model. The application *Automatic Drusen Deposits Detection in Retinal Images *(AD3RI) was developed for the validation of this methodology.

Similarly to several other works [8,11,19] only the green channel was selected for all the image processing. This channel usually offers a better drusen visibility by presenting a better contrast and less sensitivity to illumination abnormalities (when compared to the red and blue channels).

### Non-uniform illumination correction

**Figure 2:**
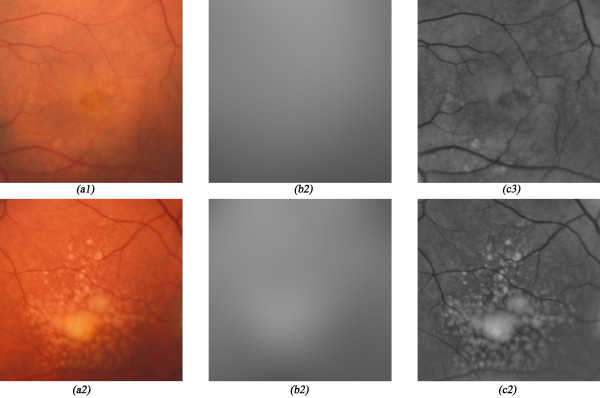
**Non-uniform illumination correction examples**. This figure shows one healthy eye retina image (*a1*) and an image of an eye containing large drusen spots (*b1*). The illumination patterns (*a2 *and *b2*) were estimated using smoothing splines. The illumination non-uniformity was corrected, dividing the original images by their illumination pattern (*a3 *and *b3*).

In ophthalmic imaging, retina pigmentation, patient's eye alignment, cataracts and optical characteristics of the fundus camera are some of the factors that can contribute to the non-uniform illumination of the acquired images (Figures 2.a1 and 2.a2)). Our proposal to solve this problem is to divide the original image by an estimation of the illumination pattern.

The estimation of the illumination pattern is obtained from the fitting of a cubic smoothing *Spline *[20] to the image *f(x,y) *(Figures 2.b1 and 2.b2)). The chosen cubic smoothing spline is a special class of *Spline *that can capture the low frequencies that characterize the non-uniformity of the illumination. The fitting objective is to minimize the equation (1).(1)

This equation contains two terms: the summation term (weighed by the smoothing factor *p*) that measures how close the spline is to the data, and the integral term (weighed by (1 - *p*)) that measures the spline smoothness using its second derivative.

The smoothing factor *p*, controls the balance between being an interpolating spline crossing all data points (with *p *= 1) and being a strictly smooth *Spline *(with *p = 0*). A too high *p *value will tend to produce, after the normalization, a flatter image, flattening also the drusen spots, which is a clearly unwanted side-effect. A too low *p *value will maintain drusen spots, but will not correct the illumination's non-uniformity.

The *p *value is also dependent on the image resolution and as resolution increases, it must also increase in order to maintain equivalent smoothness. A reference smoothing factor (*p *= 1e^-6^) was obtained empirically for a image resolution of 12.5 *μ*m/pixel. To find the relation between the smoothing factor and the image resolution, test images were resized to a predefined scale and *p *values were estimated to produce equivalent smoothing effects. The analysis of the smoothing factors showed a polynomial distribution as presented in equation (2) (take *x *as image resolution).(2)

In this fitting process, the large drusen areas influence negatively the illumination estimation, by being frequently evaluated as illumination. To overcome this, an iterative estimation process which masks drusen areas was implemented. It is based on the method proposed by Smith *et al*. [21], applied to the whole *region of interest *using two clusters.

**Figure 3:**
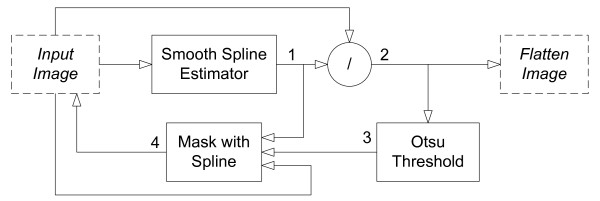
**Algorithm for the Non-Uniform Illumination correction on retinal images**. The algorithm starts by fitting a smoothing spline (1) to the input image. The input image is divided by (1), generating a flatten image (2). The approximate location of drusen spots are then estimated applying an Otsu Threshold (3) to the generated image. These areas are then replaced in the input image by the spline (1), producing a new image (4) with reduced influence of the drusen bright areas. The next iteration uses this new input image and executes the same procedure a predefined number of iterations to generate the final image.

The iterative estimation process is algorithmically described in Figure 3. It is composed by the following steps that were repeated a predefined number of times:

a. estimation of the illumination pattern (1);

b. division of the original image by the estimated illumination pattern (2);

c. binarization of the image (using Otsu thresholding) to cluster the pixels into two classes: *drusen *and *background *(3); and

d. replacement of the pixels belonging to the *drusen *class by the estimated spline in the original image, creating an image without drusen's brighter areas (4).

This process is repeated 5 times (obtained empirically) progressively reducing the influence of higher intensity pixels on the next iteration. As result of this iterative process a corrected image with uniform illumination and without lost of contrast between the background and the drusen areas is obtained (Figure 2.c1 and 2.c2)).

### Image contrast normalization

Depending on the original image contrast the non-uniform illumination correction can generate saturated or low contrasted images. This problem was corrected with the introduction of a contrast normalization procedure, achieved by normalizing the Root Mean Square contrast (*RMSc*) [22] to a predefined value. The RMS contrast calculation was based in the calculus of the contrast between retinal vessels and the background that was adopted as a reference contrast value. This calculus is applicable to any retinal image, as retinal vessels are always present.

**Figure 4:**
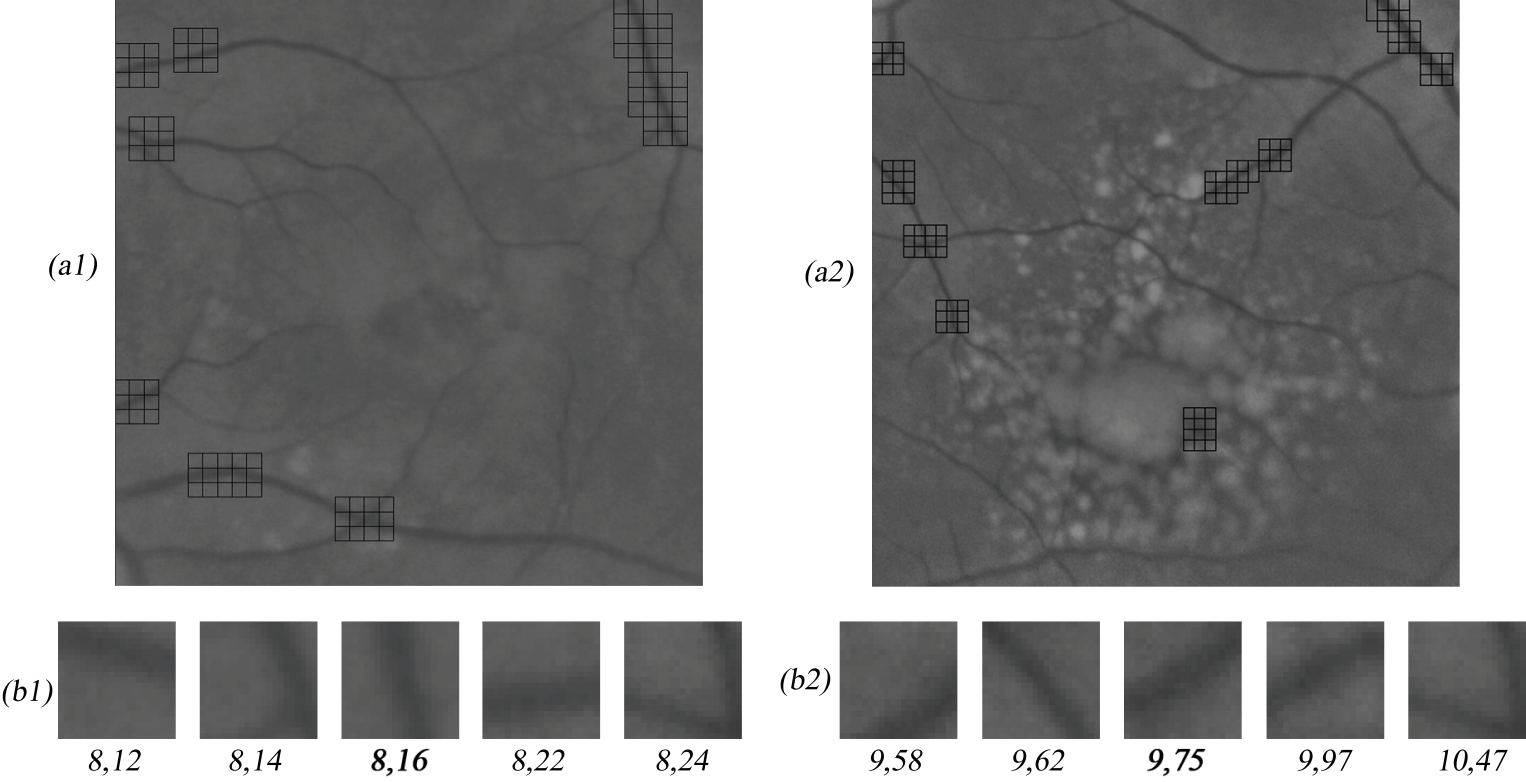
**The contrast normalization procedure**. After correcting the non-uniform illumination, the 50 darker windows are selected (*a1 *and *a2*) and the median RMS contrast of all images is considered as the overall RMS contrast value (*b1 *and *b2*).

For the vessels contrast calculation, we used a squared sliding window that locally evaluates *RMSc *contrast and mean intensity. The window was dimensioned to 250 μm wide (twice the diameter of a main vessel), in order to contain a single vessel surrounded by background. After moving the sliding window over all the image, the 51 darker windows, typically containing main vessels over a uniform background, are selected. The overall image *RMSc *- *RMSc_overall *- is calculated as the median of the 51 windows local *RMSc *(Figure 4). As the automatic algorithm can fail in some particular situations (images defects or other retinal irregularities), the window corresponding to the *RMSc_overall *value might be manually relocated by the user.

Image intensity is updated using the equation (3), with *RMSc_reference *= 15 and the constant background value A = 85. Such predefined values were obtained empirically, in order to keep 2/3 of the grey scale for representing the bright areas.(3)

### Drusen detection

**Figure 5:**
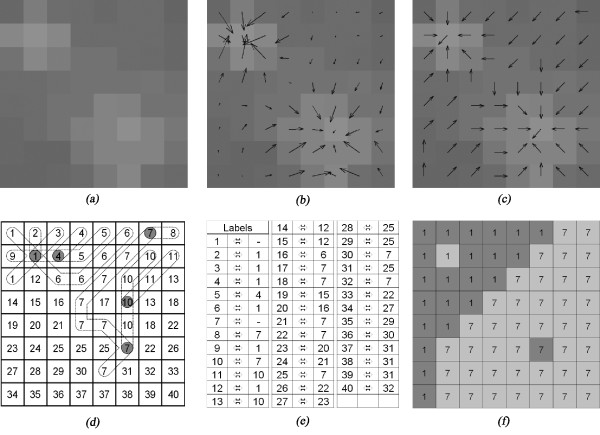
**Drusen detection algorithm example**. This figure presents a test image containing two drusen (*a*). In images (*b *and *c*) the gradient vectors and the pixels search directions are shown. Image (*d*) presents the initial *label propagation *in the two first upper rows and the label propagation paths. The labels *equivalence *table shown in figure (*e*) was built from the label propagation procedure and was applied to figure (*d*) in order to obtain the final labels image (*f*). In this example the two drusen are correctly segmented and their maximum point is highlighted.

**Figure 6:**
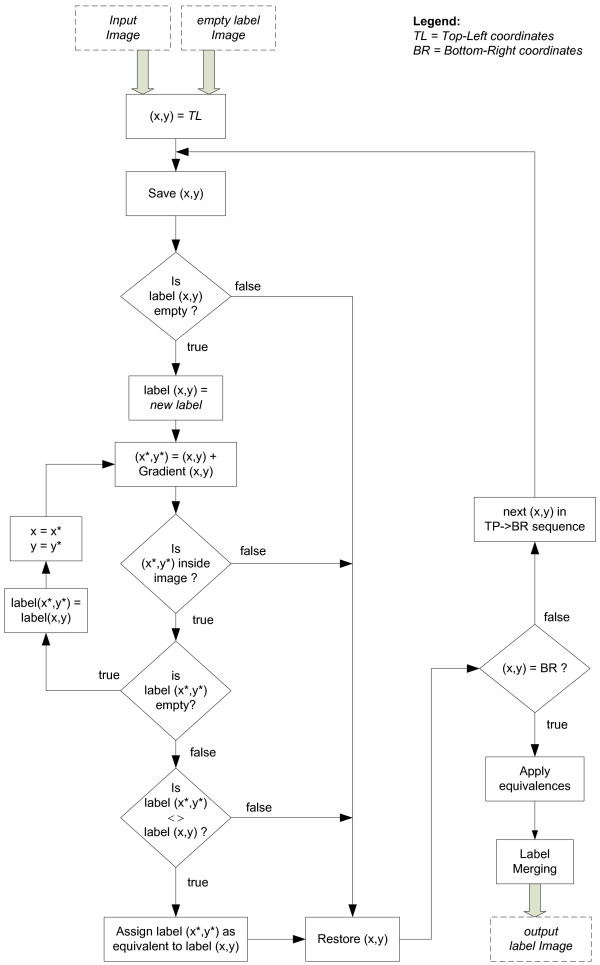
**Gradient Path Labelling Algorithm**. A step-by-step diagram of the gradient path labelling procedure is presented in this figure. The procedure receives the input image and analyses every pixel in a top-left, bottom-right direction. For every pixel it applies a *labelling *procedure, by propagating labels throughout its gradient direction and by detecting *equivalent *labels. At the end of the pixel analysis, label *equivalences *are applied in order to produce an image where each maximum is represented by a unique label. Finally, to reduce the over-segmentation produced by the algorithm, a label *merging *algorithm is applied. This merging algorithm groups adjacent regions that met the predefined amplitude merging conditions.

Drusen detection and quantification are based on the modelling of drusen to ensure shape consistency in image segmentation. The first step of the detection algorithm is to determine drusen amplitudes and locations. Considering that drusen are regional intensity maxima and that, in a gradient image, they have in its direction a confluence of several ascending paths (Figures 5.a and 5.b), the algorithm proposed for drusen detection is a novel segmentation method based on the labelling of these gradient paths (Figure 6).

The first stage of this labelling procedure is a pixel level analysis, following a top-left to bottom-right direction. It starts assigning a new label to each pixel and determining its gradient azimuth using a 3 × 3 *Sobel *operator (Figure 5.b), which is the direction to an ascending intensity. The following step, *label propagation*, propagates this label following the gradient path until an already marked or outside image boundaries pixel is found (Figures 5.c and 5.d). When the propagation process finishes on a different label, the two labels are tagged as *equivalents*, i.e., they are considered to belong to the same maximum (for example labels 2, 4, 6, 10 in Figures 5.d and 5.e).

The second stage of the labelling procedure is to *apply the equivalences*. *Equivalent *labels are grouped and replaced on the image by the smaller one of each group (Figure 5.f) producing a segmented image with as many labels as drusen spots.

When flat valleys or flat hills exist, not all gradient paths end on the same maximum pixel, resulting in an over-segmentation of the image. To solve this problem, a *merging *algorithm was introduced as the last stage of the labelling procedure.

**Figure 7:**
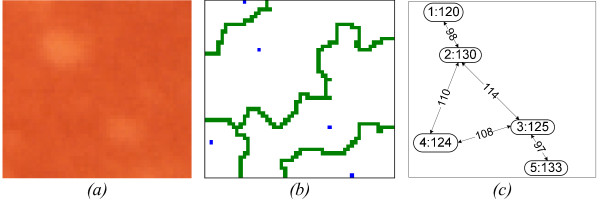
**Merging Algorithm**. This figure shows the segmentation *(b) *of a small region of a retina image *(a) *and its corresponding connectivity graph *(c)*. The connectivity graph represents the connections between segmentation areas and is labelled with the minimum intensity border value on each *link*. Each *node *contains the label and the intensity maximum of the segmentation area it represents.

Analysing the labelled and the original image, the *merging *algorithm begins by creating a graph where *nodes *correspond to labels and *links *represent the adjacencies between them (Figure 7). Each *node *is characterized by the maximum intensity level of the pixels in its region and each *link *is set with the minimum intensity value of the border pixels between the two adjacent regions. If the difference between the *link *and the *nodes *is below a predefined threshold (*Δ_a_*), the two corresponding regions are merged. The value of *Δ_a _*adjusts the detail of the analysis (*Δ_a _= 3 *was empirically found to be adequate for our normalized images).

After the *merging*, drusen are segmented and characterized by the coordinates and amplitude of their maximum intensity, which are the initial parameters for the modelling algorithm.

### Drusen modelling

**Figure 8:**
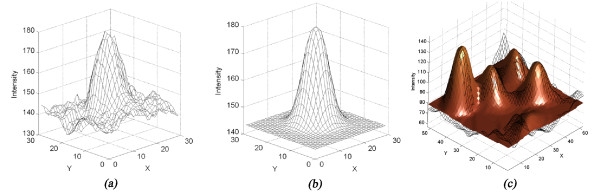
**Drusen modelling**. The similarity between the three dimensional view of a druse (*a*) and a Gaussian shape (*b*) is shown. Figure (*c*) shows the final result of the modelling procedure for an image containing superimposed drusen (*grid *- original image; *surface *- image model).

In order to quantify drusen spots it is necessary to analytical characterize their shape and intensity. The intensity elevations shown on drusen areas on the tri-dimensional representation (Figure 8.a) motivated the creation of a model of the image intensity. From this model the drusen dimensions and the total affected area are extracted.

### Modelling function

In a typical three-dimensional view of a druse (Figure 8.a), it can be seen that it has a shape similar to a Gaussian function (Figure 8.b). Based on this observation, the Modified Gaussian function (equation (4)) was adopted to individually model the drusen spots. This function allows translations in the *xyz *axes (*x*_0_, *y*_0, _*z_0_*), amplitude scaling (*A*), rotation (*θ*) and shape adjustments (*σ_x_*, *s_F_*, *d*). These latter define the width in the *x*-plane (*σ_x_*); width in *y*-plane (*s_F _*); and the amplitude profile between square shape, bell shape and thin shape (*d *).(4)

The Levenberg-Marquardt Least-Squares optimization algorithm [23] was used to fit the multiple elementary functions to the image (Figure 8.c) adjusting the functions parameters in order to minimize the mean square error between the model and the image. The algorithm was improved by including interval constraints in the amplitude and shape factor parameters, in order to guarantee the convergence of the solution and reduce computation time.

### Image sectioning

The modelling of the image by multiple functions, each containing eight adjustable parameters, is time consuming and can be non-convergent. To reduce complexity and improve convergence, an image sectioning method was implemented. Its goal was to create smaller images containing isolated or confluent drusen to be processed individually.

The sectioning process begins by applying a threshold to the normalized image (10% above the normalized background). The result is an image where drusen (isolated or confluent) are roughly identified and surrounded by background. The process is followed by a *connected components *object detection algorithm [4] to identify and label the drusen areas. Finally, these marked areas are copied from the original image to new smaller images containing just the identified drusen surrounded by background. These small images are then individually analysed by the modelling algorithm, requiring lower complexity.

### Drusen Area Quantification

The contour of drusen spots and their area are calculated by thresholding the analytical model. The threshold value, that produces more accurate contours, was determined by comparing the false-positives and the false-negative pixels between the automated method and all the manually graded images. The threshold value is defined as a percentage of the background value used for the image normalization.

**Figure 9:**
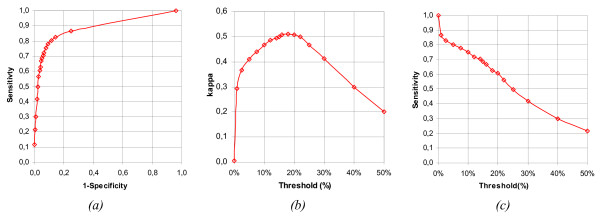
**Algorithm sensitivity for different parameterizations**. This figure represents sensitivity, specificity and *kappa *coefficient values obtained by the automated method using different threshold values when compared to the ground truth. The results are presented as a ROC curve *(a)*, a plot of the *kappa *coefficient vs. threshold *(b) *and a plot of sensitivity vs. threshold *(c)*. The optimal threshold value was considered to be at approximately 18%, corresponding to the maximum of the *kappa *coefficient.

The threshold value was found by comparing the statistics for different threshold values (between 0% and 50%) by plotting their Receiver Operating Characteristic curve (ROC) and Cohen's *kappa *Coefficient Curve (Figure 9). The curves show maximum accuracy at a threshold of 18% with a *kappa *coefficient of 0.51, a sensitivity of 0.63 and a specificity of 0.96. The 18% threshold was therefore adopted as the quantification threshold.

### Validation

To validate and assess the accuracy of the automated method (AD3RI) it was compared to the gradings done by the experts and to a classical Global Threshold method. The Global Threshold method was applied to the normalized images and used an empirically found threshold value of 30% of the background value used for the image normalization.

For evaluation purposes it was assumed that a good performance of the AD3RI would be to obtain an overall score similar to the obtained by the experts. Experts were also evaluated among themselves, in order to produce an efficiency score for each of them.

AD3RI, Experts and Threshold gradings were assessed using both overall and local agreement indicators. Based on the total affected area, two overall indicators were used: the Coefficient of Variation (CV) and the Intraclass Correlation Coefficient (ICC). Local indicators sensitivity, specificity and *kappa *coefficient were based on a pixel-to-pixel analysis that determines false-positive and false-negative pixels.

## Results and discussion

**Figure 10:**
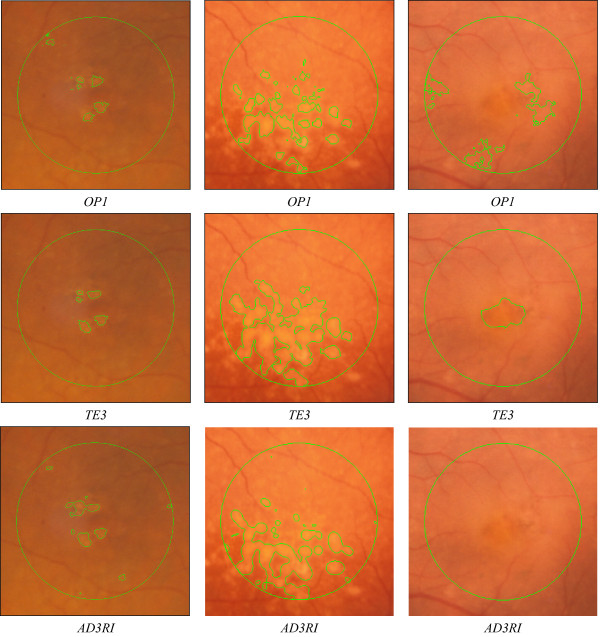
**Grading examples**. This figure shows, from left to right, images #1, #20 and #22, graded by different experts (first and second row) and by AD3RI (third row). A high agreement was obtained among the expert's gradings of the two images represented on the left side and that show normal drusen spots. On the right side it is shown a control image containing no visible drusen that was considered an outlier due to the high variability observed among the experts.

The validation of the methodology was made with the dataset of twenty two retinal images. A first examination of the total affected areas, as presented in Table 1, showed a significant variability within the experts' gradings (43% average). There was a poor agreement among them especially on some low contrast images, creating an issue for image comparison purposes. User's subjectivity and different contrast and illumination settings were the main causes for the different analysis criteria. To illustrate these differences, Figure 10 presents three images processed by the AD3RI side-by-side with the experts' gradings. It can be seen that, on the right image, which was the one with highest variability, there was almost no agreement on drusen sizes and locations. As a result of this variability, an outlier identification policy was implemented. Therefore, the images which had a CV among the experts above 50% (#8, #10, #11, #18 and #22) were considered outliers and excluded from the study.

**Table 1:** Automatic vs. manual measurements of Drusen per image

*#*	OP1	OP2	OP3	OP4	TE1	TE2	TE3	TE4	AD3RI	CV_Experts_	CV_AD3RI_
**1**	1.8	2.2	2.0	2.2	1.3	1.3	1.6	2.0	3.0	21%	63%
**2**	3.2	4.4	3.3	4.2	2.9	3.4	2.7	6.2	1.8	30%	52%
**3**	8.3	4.4	5.8	7.8	6.9	4.3	6.1	7.1	7.4	23%	17%
**4**	2.7	1.3	0.9	1.2	1.2	1.5	1.5	0.6	1.7	46%	24%
**5**	4.9	3.7	4.3	5.0	2.0	3.5	5.0	5.7	5.8	27%	36%
**6**	10.7	5.8	7.6	10.7	10.2	9.1	10.0	18.3	11.9	36%	15%
**7**	13.0	9.5	9.7	18.1	10.3	14.4	12.3	22.8	14.0	34%	2%
*8	10.1	3.3	0.1	4.5	3.6	3.0	2.2	9.5	5.2	77%	14%
**9**	13.8	13.8	14.6	20.2	15.1	3.9	20.6	31.7	12.9	48%	23%
*10	7.7	1.8	4.2	2.5	7.6	4.9	10.2	12.0	10.7	57%	68%
*11	2.1	3.9	7.5	17.8	20.3	5.3	20.1	12.1	11.4	67%	2%
**12**	5.9	5.5	6.7	6.1	6.7	6.6	8.3	7.2	5.6	13%	15%
**13**	1.2	3.4	2.3	2.3	2.9	2.2	3.7	2.7	1.4	30%	47%
**14**	3.5	3.6	3.6	5.6	3.8	4.5	6.5	3.5	5.3	26%	23%
**15**	1.4	5.2	8.4	6.7	5.7	6.6	6.4	7.3	6.1	35%	3%
**16**	1.9	5.3	4.3	4.8	3.4	4.0	4.5	5.9	4.8	29%	12%
**17**	1.2	1.6	1.1	0.4	0.8	1.3	1.9	1.4	2.6	38%	113%
*18	1.0	1.1	2.0	0.2	0.8	1.4	1.7	4.1	1.2	77%	19%
**19**	31.0	25.2	51.0	45.5	44.9	41.7	55.9	63.2	52.2	28%	16%
**20**	11.7	16.2	18.4	20.5	17.0	15.5	16.7	27.2	19.3	25%	8%
**21**	9.3	15.8	9.1	7.1	6.7	11.2	14.2	16.1	15.1	34%	35%
*22	12.5	7.3	4.6	0.7	0.1	0.0	0.0	0.8	0.0	141%	100%

This table shows the measurements of drusen areas as a percentage of the total macular area for each expert and for AD3RI. It is also shown the CV among experts and the CV of the automated method when compared to the ground truth for the twenty two test images. As it can be seen in the outlier images signed with an asterisk (*), the CV among experts was significant (> 50%), especially in image #22 where only 3 out of 8 experts graded significant drusen areas. It should also be noticed that no significant differences in the results produced by the group of Ophthalmologists (OP) and by the group of Technicians (TE) were observed. In this table it can be observed that AD3RI obtained a good accuracy with a CV lower than the average CV of the experts in 10 out of 17 images (dataset without outliers). In the remaining 7 images AD3RI obtained a higher CV mainly due to the small drusen areas in which even a small difference in the area value penalizes significantly its CV.

Table 2 presents the summary of results obtained by the AD3RI, the Global Threshold method and each expert, excluding the outlier images. Areas' comparison (CV and ICC) showed that, although the CV obtained by the AD3RI (28.8%) was above the average among the experts (22.5%), the ICC (0.92) revealed a strong correlation between AD3RI and the experts. The images containing few drusen spots were the main cause for a higher CV. In these images, the total affected area is low and an over or under estimation of drusen spots will cause a significant relative variation on the total area, increasing its CV. The Threshold method showed a low agreement, obtaining a high CV (41.1%) and a low correlation (ICC = 0.67).

**Table 2:** Summary of average indicators for automatic and manual measurements

	CV	ICC	Sensitivity	Specificity	Kappa
**AD3RI**	28,8%	0,92	0,68	0,96	0,58
**THRESH**	41,1%	0,67	0,74	0,94	0,49
**OP1**	28,3%	0,86	0,58	0,98	0,55
**OP2**	23,6%	0,79	0,66	0,98	0,61
**OP3**	15,0%	0,92	0,69	0,97	0,64
**OP4**	19,6%	0,92	0,61	0,96	0,53
**TE1**	16,3%	0,92	0,66	0,98	0,64
**TE2**	14,2%	0,89	0,65	0,98	0,62
**TE3**	21,9%	0,90	0,76	0,96	0,64
**TE4**	41,2%	0,82	0,77	0,93	0,57

This table presents the average CV, ICC, sensitivity, specificity and *kappa *coefficient obtained by AD3RI and by the Thresholding method compared to the manual gradings done by each of the experts (OP - Ophthalmologist; TE - Technician). These results exclude the images considered as outliers. As it can be seen, AD3RI and manual gradings obtained similar values; only for CV the AD3RI results were above the average. This is justified by the level of detail of the AD3RI analysis that is usually superior to the manual analysis. AD3RI obtained better results than the Thresholding method on all the indicators except on sensitivity. However, Thresholding sensitivity is higher due to its over-detection of drusen what consequently decreases its performance on the other indicators.

When examining the accuracy on a pixel-to-pixel comparison, the AD3RI achieved an average sensitivity of 0.68 and an average specificity of 0.96, while the experts obtained 0.67 and 0.97. The slightly lower specificity obtained by the AD3RI was mainly due to the higher detection of drusen as consequence of a more detailed and systematic analysis. The *kappa *coefficient, analyzed accordingly to Landis and Koch guidelines [24], showed a *moderate *agreement for both AD3RI (*k *= 0.58) and experts average (*k *= 0.60).

The Threshold method showed a sensitivity of 0.74, higher than the average obtained by the experts as consequence of an over-detection of drusen. However this over-detection penalizes significantly its specificity (0.94) and its *kappa *coefficient (0.49).

From this statistical analysis it was concluded that the proposed algorithm follows the same criteria as the experts, although with a better accuracy and reproducibility.

The Global Threshold method showed a low agreement with the experts. Comparing Thresholding with AD3RI gradings, it can be observed that AD3RI, although with less detailed contours, has lower illumination dependency and provides more consistent drusen shape segmentation with higher reproducibility. The Threshold method is a simpler method, but shows an important tendency for drusen over-detection, producing a higher number of false-positives.

The analysis of the related work shows a large number of different methods and indicators for performance evaluation, limiting the comparison with our method. In the work of Rapantzikos *et al*. [8] their algorithm was tested in a set of twenty three images and compared to two experts analyses. For the specificity and sensitivity analysis the interception between the experts' gradings was used. This methodology decreased the probability of false-negatives, consequently rising sensitivity. These two indicators exceeded 0.96 in all cases, which can be considered excellent. However, it should be noted that the experts' interception is not a reliable method, since it eliminates variability, increasing sensitivity without compromising specificity. Smith *et al*. [5] evaluated their work with a dataset of twenty images examined by one expert, obtaining a sensitivity of 0.7 and a specificity of 0.8. Therefore, we can consider AD3RI more accurate namely because it was tested against a set of eight experts in order to achieve a more reliable ground-truth.

## Conclusions

The development of methods to quantitatively measure drusen in a reproducible and accurate procedure will certainly improve the quality of the follow up of this disease and potentiate epidemiologic studies and clinical trials. These studies, that collect thousands of images throughout several years, must be graded using a reproducible method to allow comparison during all the study period. Currently, this is manually done by trained experts with a fastidious process, lacking accuracy and reproducibility.

This article presents a new method to quantitatively measure drusen and its' validation with 22 images graded by eight independent experts. The algorithm is based on the detection and modelling of drusen to automatically calculate the affected areas. It includes also an image pre-processing step to correct the non-uniform illumination commonly found on this type of images.

The illumination compensation algorithm is an important step to obtain a less parameterized methodology, since it is capable to create an image with normalized illumination and contrast to be used in all the remaining steps. The detection and modelling of drusen with Modified Gaussian functions demonstrated its capability to segment drusen keeping their typical shape, even on low contrast images.

It also provides an analytical model that allows the determination of drusen indicators such as number of spots, affected areas, confluence and average size.

Since there is no standard assessment technique to be applied in this type of studies, most of the published works use different performance indicators what makes comparison between studies inaccurate or even impossible. In our work, performance was assessed using several indicators allowing direct comparison with other studies. This comparison showed that the results produced by the AD3RI were similar or better than the others.

From the above, we considered that AD3RI demonstrated promising results. It compares positively with the panel of human experts and since is a determinist method; it is not dependent on factors such as attention or accuracy.

## Competing interests

The authors declare that they have no competing interests.

## Authors' contributions

ADM carried out the study design, the statistical analysis and drafted the manuscript. PMV, JMF and AM supervised the study design, helped in the results analysis and reviewed the manuscript.

All authors read and approved the final manuscript.
